# 5-[(*Z*)-2,3-Dimeth­oxy­benzyl­idene]-1,2,4-triazolo[3,2-*b*][1,3]thia­zol-6(5*H*)-one

**DOI:** 10.1107/S1600536812042559

**Published:** 2012-10-20

**Authors:** Lu Guo, Gao-Tong Lin, Jia Wang, Li Ni, Ren-Shan Ge

**Affiliations:** a Wenzhou Medical College, School of Pharmacy, Wenzhou 325035, People’s Republic of China

## Abstract

The title compound, C_13_H_11_N_3_O_3_S, was synthesized from 1*H*-1,2,4-triazole-5-thiol in a one pot reaction. The fused thia­zolo[3,2-*b*][1,2,4]triazole system is essentially coplanar with the benzene ring: they enclose an inter­planar angle of 1.37 (13)°. The olefinic double bond is in a *Z* conformation. In the crystal, C—H⋯N hydrogen bonds link the mol­ecules into double layers parallel to the *ab* plane.

## Related literature

For related structures, see: Özbey *et al.* (1999 ▶); Köysal *et al.* (2004 ▶). For background to the biological properties of fused thia­zolo[3,2-*b*][1,2,4]triazol derivatives, see: El-Sherif *et al.* (2006 ▶); Gilbertsen *et al.* (1999 ▶); Karthikeyan (2009 ▶); Lesyk *et al.* (2007 ▶); Martin *et al.* (1999 ▶); Tozkoparan *et al.* (2000 ▶, 2002 ▶, 2007 ▶).
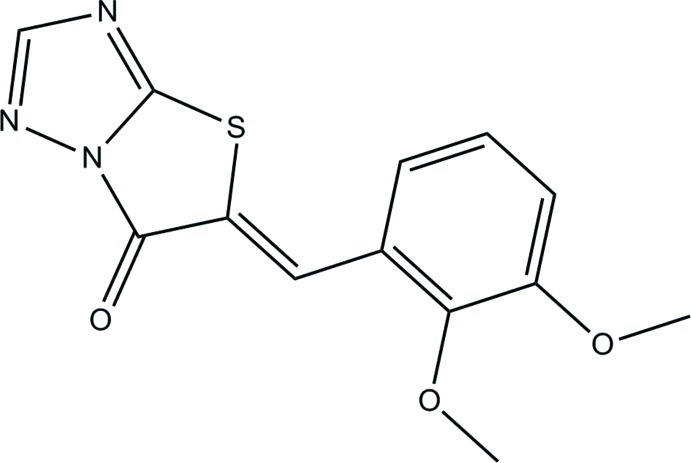


## Experimental

### 

#### Crystal data

C_13_H_11_N_3_O_3_S
*M* *_r_* = 289.31
Monoclinic, 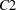

*a* = 11.5904 (13) Å
*b* = 7.0570 (8) Å
*c* = 16.4519 (18) Å
β = 107.445 (2)°
*V* = 1283.8 (2) Å^3^
*Z* = 4
Mo *K*α radiation
μ = 0.26 mm^−1^
*T* = 293 K
0.32 × 0.22 × 0.20 mm

#### Data collection

Bruker SMART CCD area-detector diffractometer
Absorption correction: multi-scan (*SADABS*; Bruker, 2002 ▶) *T*_min_ = 0.314, *T*_max_ = 1.000
3567 measured reflections
2096 independent reflections
2022 reflections with *I* > 2σ(*I*)
*R*_int_ = 0.074

#### Refinement

*R*[*F* ^2^ > 2σ(*F* ^2^)] = 0.050
*wR*(*F* ^2^) = 0.135
*S* = 1.07
2096 reflections
184 parameters
1 restraint
H-atom parameters constrained
Δρ_max_ = 0.30 e Å^−3^
Δρ_min_ = −0.40 e Å^−3^
Absolute structure: Flack (1983 ▶), 724 Friedel pairs
Flack parameter: 0.00 (10)

### 

Data collection: *SMART* (Bruker, 2002 ▶); cell refinement: *SAINT* (Bruker, 2002 ▶); data reduction: *SAINT*; program(s) used to solve structure: *SHELXS97* (Sheldrick, 2008 ▶); program(s) used to refine structure: *SHELXL97* (Sheldrick, 2008 ▶); molecular graphics: *SHELXTL* (Sheldrick, 2008 ▶); software used to prepare material for publication: *SHELXTL*.

## Supplementary Material

Crystal structure: contains datablock(s) I, global. DOI: 10.1107/S1600536812042559/fy2070sup1.cif

Structure factors: contains datablock(s) I. DOI: 10.1107/S1600536812042559/fy2070Isup2.hkl

Supplementary material file. DOI: 10.1107/S1600536812042559/fy2070Isup3.cml

Additional supplementary materials:  crystallographic information; 3D view; checkCIF report

## Figures and Tables

**Table 1:** Hydrogen-bond geometry (Å, °)

*D*—H⋯*A*	*D*—H	H⋯*A*	*D*⋯*A*	*D*—H⋯*A*
C9—H9⋯N2^i^	0.93	2.46	3.375 (4)	167
C8—H8⋯N3^ii^	0.93	2.60	3.529 (4)	173
C1—H1⋯N3^iii^	0.93	2.65	3.556 (4)	164

Symmetry codes: (i) 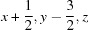; (ii) 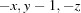; (iii) 
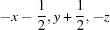
.

**Fig. 1.**
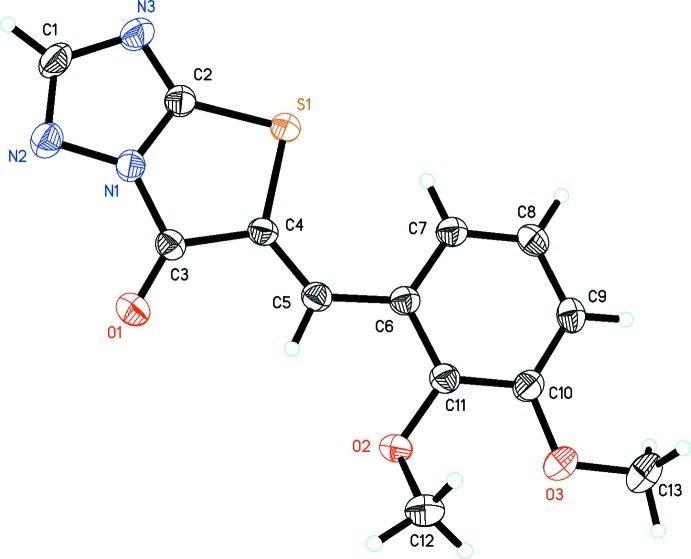
Molecular structure of the title compound, showing 30% displacement ellipsoids for the non-hydrogen atoms. Hydrogen atoms are drawn as spheres of arbitrary radius.

**Fig. 2.**
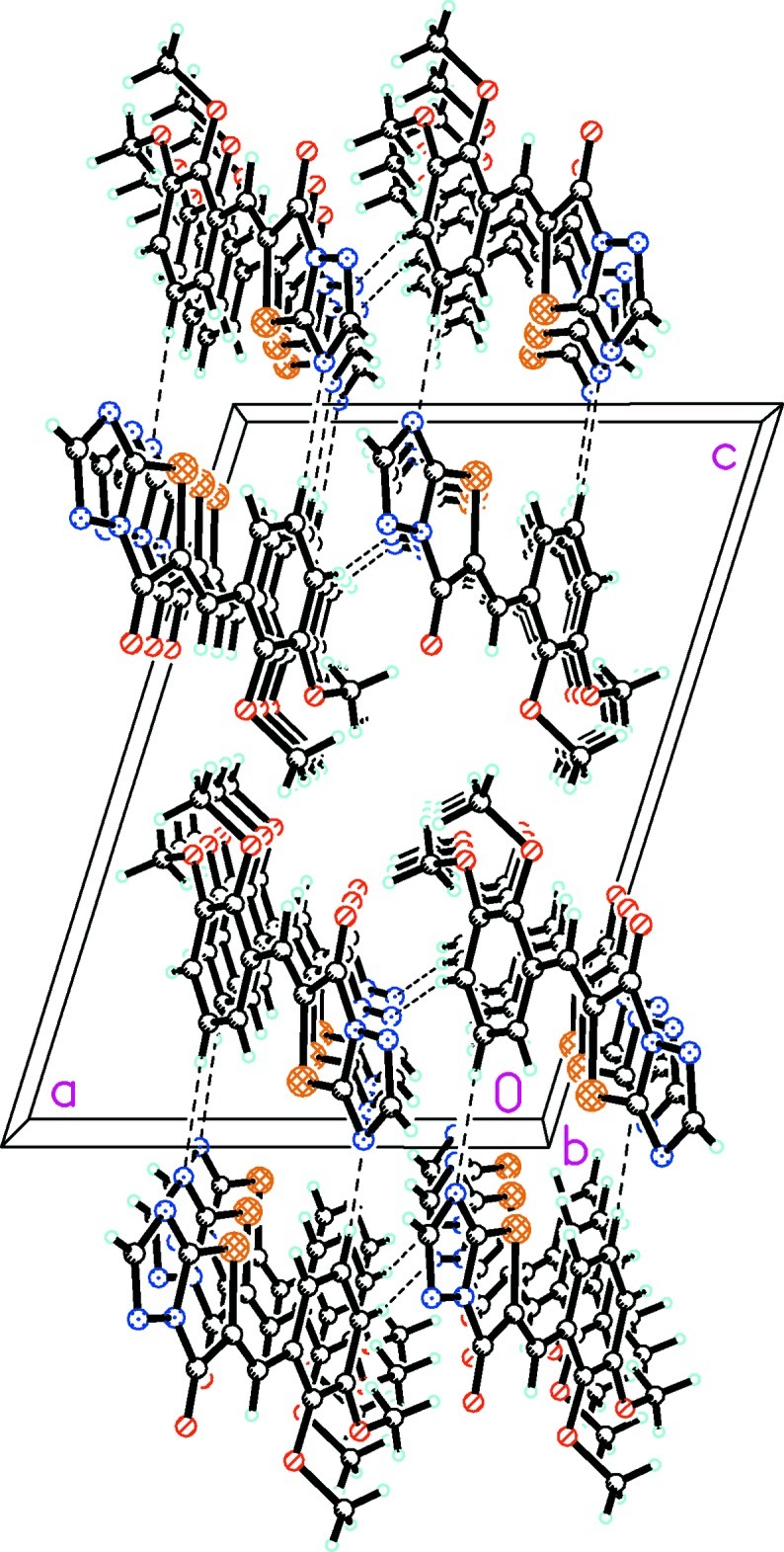
A crystal-packing diagram the title compound. Hydrogen bonds are shown as dashed lines.

## supplementary crystallographic information

### Comment

Darbufelone, a 5-(3,5-di-*tert*-butyl-4-hydroxybenzylidene)thiazol-4-one derivative, is a new non-steroidal anti-inflammatory drug (NSAID), acting as a COX-2 and LOX-5 dual inhibitor (Martin *et al.*, 1999). It is made and developed by the Warner-Lambert Company of the USA. The title compound, 5(*Z*)-(2,3-dimethoxybenzylidene)thiazolo[3,2-*b*][1,2,4]triazol-6(5*H*)-one, is structurally related to Darbufelone and other compounds bearing fused thiazole and triazole rings, which have attracted our interest in a search for better anti-inflammatory agents.

The thiazole and 1,2,4-triazole moieties are present in various molecules having biological activity, especially in antiinflammatory agents (Karthikeyan, 2009; Tozkoparan *et al.*, 2002, 2007). Compounds which contain fused thiazole and triazole rings, thiazolo-triazoles, also show significant biological and pharmacological properties: antiinflammatory, antitumoral, analgesic, antipyretic, antimicrobial (Tozkoparan *et al.*, 2000; Lesyk *et al.*, 2007; El-Sherif *et al.*, 2006). Thus, in the present work, with the aim of further clarifying the molecular structure of this type of compounds, the single-crystal X-ray analysis of 5(*Z*)-(2,3-dimethoxybenzylidene)thiazolo[3,2-*b*][1,2,4]triazol-6(5*H*)-one(I), has been carried out.

The title compound consists of a fused thiazolo[3,2-*b*]-l,2,4-triazole system and a phenyl group which bears 2,3-dimethoxy substituents (Fig. 1). The thiazolo[3,2-*b*][1,2,4]triazole system is essentially planar and the two rings nearly share a common plane with interplanar angle of 0.89 (18)°. The r.m.s. deviation of heavy atoms from the triazole and thiazolo ring mean planes are 0.0034 and 0.0020 Å, respectively. The benzene ring at the C5 position is in the *cis* (*Z*) configuration. The C4—C5 bond is a double bond [1.343 (4) Å]. The C3—C4 [1.489 (4), Å,] and C5—C6 [1.439 (4) Å] bonds are found to have normal single-bond lengths. A similar lengthening of the S1—C2 bond relative to the S1—C4 bond has been observed in the structure of a similar compound (Özbey *et al.*, 1999; Köysal *et al.*, 2004). The crystal of the title compound is stabilized by C—H···N intermolecular contacts. There are three intermolecular hydrogen-bond interactions: C9—H9···N2, C8—H8···N3 and C1—H1···N3 (Table 1).

### Experimental

4 ml of 90% formic acid contained in a round-bottomed flask was heated at 100°C for 15 minutes, and then 1.82 g (20 mmol) thiosemicarbazide was added. The heating was continued for 30 minutes, during which time crystalline 2-formylhydrazinecarbothioamide separated. The reaction mixture was cooled to 0 °C to give a white solid. Then a solution of 10 mmol 2-formylhydrazinecarbothioamide and 2 ml 20% NaOH was heated for 1 h. The reaction mixture was cooled, poured onto crushed ice, and neutralized with conc. HCl. The resulting solid 1*H*-1,2,4-triazole-5-thiol was filtered, dried, and recrystallized from ethanol.

To 1 mmol of 1*H*-1,2,4-triazole-5-thiol, 1.5 mmol of monochloroacetic acid, 0.01 mol of 2,3-dimethoxy benzaldehyde, 1 ml acetic anhydride, 0.01 mol of anhydrous sodium acetate, and 2 ml glacial acetic acid were added and refluxed for 3 h. The reaction mixture was cooled, and poured onto crushed ice. The mixture was then allowed to reach room temperature, then filtered and washed with water to obtain a crude product. The resulting solid was collected and crystallized from acetic acid. Single crystals were grown from acetic acid by slow evaporation.

### Refinement

The H atoms were positioned geometrically (C—H = 0.93 and 0.96 Å) and refined as riding with *U*_ĩso_(H) = 1.2*U*_eq_(C) or 1.5*U*_eq_(methyl C).

### Crystal data

**Table:**

C_13_H_11_N_3_O_3_S	*F*(000) = 600
*M**_r_* = 289.31	*D*_x_ = 1.497 Mg m^−^^3^
Monoclinic, *C*2	Mo *K*α radiation, λ = 0.71073 Å
*a* = 11.5904 (13) Å	Cell parameters from 2670 reflections
*b* = 7.0570 (8) Å	θ = 5.2–55.1°
*c* = 16.4519 (18) Å	µ = 0.26 mm^−^^1^
β = 107.445 (2)°	*T* = 293 K
*V* = 1283.8 (2) Å^3^	Prismatic, green
*Z* = 4	0.32 × 0.22 × 0.20 mm

### Data collection

**Table:**

Bruker SMART CCD area-detector diffractometer	2096 independent reflections
Radiation source: fine-focus sealed tube	2022 reflections with *I* > 2σ(*I*)
Graphite monochromator	*R*_int_ = 0.074
φ and ω scans	θ_max_ = 26.0°, θ_min_ = 2.6°
Absorption correction: multi-scan (*SADABS*; Bruker, 2002)	*h* = −12→14
*T*_min_ = 0.314, *T*_max_ = 1.000	*k* = −6→8
3567 measured reflections	*l* = −20→17

### Refinement

**Table:**

Refinement on *F*^2^	Hydrogen site location: inferred from neighbouring sites
Least-squares matrix: full	H-atom parameters constrained
*R*[*F*^2^ > 2σ(*F*^2^)] = 0.050	*w* = 1/[σ^2^(*F*_o_^2^) + (0.102*P*)^2^] where *P* = (*F*_o_^2^ + 2*F*_c_^2^)/3
*wR*(*F*^2^) = 0.135	(Δ/σ)_max_ < 0.001
*S* = 1.07	Δρ_max_ = 0.30 e Å^−^^3^
2096 reflections	Δρ_min_ = −0.40 e Å^−^^3^
184 parameters	Extinction correction: *SHELXL*, Fc^*^=kFc[1+0.001xFc^2^λ^3^/sin(2θ)]^-1/4^
1 restraint	Extinction coefficient: 0.004
Primary atom site location: structure-invariant direct methods	Absolute structure: Flack (1983), 724 Friedel pairs
Secondary atom site location: difference Fourier map	Flack parameter: 0.00 (10)

### Special details

**Table:**

Geometry. All e.s.d.'s (except the e.s.d. in the dihedral angle between two l.s. planes) are estimated using the full covariance matrix. The cell e.s.d.'s are taken into account individually in the estimation of e.s.d.'s in distances, angles and torsion angles; correlations between e.s.d.'s in cell parameters are only used when they are defined by crystal symmetry. An approximate (isotropic) treatment of cell e.s.d.'s is used for estimating e.s.d.'s involving l.s. planes.
Refinement. Refinement of *F*^2^ against ALL reflections. The weighted *R*-factor *wR* and goodness of fit *S* are based on *F*^2^, conventional *R*-factors *R* are based on *F*, with *F* set to zero for negative *F*^2^. The threshold expression of *F*^2^ > σ(*F*^2^) is used only for calculating *R*-factors(gt) *etc*. and is not relevant to the choice of reflections for refinement. *R*-factors based on *F*^2^ are statistically about twice as large as those based on *F*, and *R*- factors based on ALL data will be even larger.

### Fractional atomic coordinates and isotropic or equivalent isotropic displacement parameters (Å^2^)

**Table:**

	*x*	*y*	*z*	*U*_iso_*/*U*_eq_	
S1	−0.01455 (7)	1.05191 (12)	0.10793 (4)	0.0509 (3)	
N1	−0.0780 (2)	1.3372 (4)	0.18332 (14)	0.0416 (5)	
N2	−0.1377 (2)	1.5049 (4)	0.17612 (17)	0.0508 (7)	
N3	−0.1513 (3)	1.3773 (5)	0.04552 (16)	0.0590 (8)	
O1	0.00045 (19)	1.2734 (4)	0.32636 (12)	0.0554 (6)	
O2	0.21278 (18)	0.7058 (4)	0.40934 (11)	0.0527 (6)	
O3	0.3203 (2)	0.3716 (4)	0.39185 (15)	0.0661 (7)	
C1	−0.1790 (3)	1.5220 (5)	0.0926 (2)	0.0568 (9)	
H1	−0.2245	1.6262	0.0669	0.068*	
C2	−0.0877 (3)	1.2682 (5)	0.10527 (16)	0.0464 (7)	
C3	−0.0130 (2)	1.2290 (4)	0.25439 (15)	0.0389 (6)	
C4	0.0302 (2)	1.0558 (5)	0.22076 (14)	0.0380 (6)	
C5	0.0954 (2)	0.9259 (4)	0.27501 (16)	0.0395 (6)	
H5	0.1093	0.9551	0.3323	0.047*	
C6	0.1475 (2)	0.7493 (5)	0.25979 (16)	0.0390 (6)	
C7	0.1392 (3)	0.6801 (5)	0.17817 (17)	0.0441 (7)	
H7	0.0975	0.7497	0.1304	0.053*	
C8	0.1919 (3)	0.5107 (5)	0.16797 (18)	0.0468 (7)	
H8	0.1847	0.4659	0.1135	0.056*	
C9	0.2555 (3)	0.4070 (5)	0.23833 (19)	0.0464 (7)	
H9	0.2930	0.2946	0.2308	0.056*	
C10	0.2643 (2)	0.4684 (5)	0.32006 (18)	0.0455 (7)	
C11	0.2112 (2)	0.6410 (5)	0.33035 (17)	0.0416 (6)	
C12	0.3298 (3)	0.7237 (7)	0.47229 (19)	0.0620 (10)	
H12A	0.3520	0.6048	0.5011	0.093*	
H12B	0.3262	0.8195	0.5129	0.093*	
H12C	0.3889	0.7585	0.4447	0.093*	
C13	0.3783 (4)	0.1988 (7)	0.3845 (3)	0.0700 (10)	
H13A	0.3209	0.1138	0.3482	0.105*	
H13B	0.4105	0.1427	0.4399	0.105*	
H13C	0.4429	0.2225	0.3606	0.105*	

### Atomic displacement parameters (Å^2^)

**Table:**

	*U*^11^	*U*^22^	*U*^33^	*U*^12^	*U*^13^	*U*^23^
S1	0.0663 (5)	0.0506 (5)	0.0329 (3)	0.0151 (4)	0.0104 (3)	−0.0014 (3)
N1	0.0420 (11)	0.0388 (14)	0.0433 (11)	0.0040 (10)	0.0117 (9)	−0.0009 (10)
N2	0.0488 (13)	0.0465 (18)	0.0533 (13)	0.0060 (12)	0.0095 (10)	0.0010 (12)
N3	0.0694 (16)	0.0561 (19)	0.0425 (13)	0.0161 (16)	0.0031 (12)	0.0062 (13)
O1	0.0653 (13)	0.0592 (16)	0.0403 (10)	0.0085 (12)	0.0136 (9)	−0.0072 (11)
O2	0.0555 (11)	0.0690 (17)	0.0331 (9)	0.0154 (11)	0.0126 (8)	0.0032 (10)
O3	0.0809 (15)	0.0590 (18)	0.0581 (13)	0.0246 (13)	0.0205 (11)	0.0176 (12)
C1	0.0598 (16)	0.047 (2)	0.0556 (16)	0.0153 (16)	0.0057 (14)	0.0117 (16)
C2	0.0492 (14)	0.0461 (17)	0.0409 (13)	0.0050 (14)	0.0090 (11)	−0.0015 (13)
C3	0.0373 (12)	0.0418 (17)	0.0376 (12)	−0.0009 (12)	0.0113 (10)	−0.0036 (12)
C4	0.0377 (11)	0.0444 (16)	0.0315 (10)	0.0002 (13)	0.0096 (9)	−0.0006 (13)
C5	0.0381 (11)	0.0444 (17)	0.0367 (12)	0.0002 (12)	0.0125 (10)	0.0006 (12)
C6	0.0366 (11)	0.0429 (16)	0.0376 (12)	−0.0013 (12)	0.0112 (10)	0.0020 (12)
C7	0.0462 (14)	0.0458 (17)	0.0387 (12)	0.0012 (13)	0.0102 (11)	0.0008 (13)
C8	0.0521 (14)	0.047 (2)	0.0433 (13)	−0.0039 (14)	0.0167 (11)	−0.0081 (13)
C9	0.0473 (14)	0.0385 (16)	0.0573 (16)	0.0006 (13)	0.0217 (12)	−0.0038 (14)
C10	0.0426 (13)	0.0462 (17)	0.0478 (15)	0.0039 (13)	0.0136 (12)	0.0076 (12)
C11	0.0395 (12)	0.0473 (17)	0.0395 (13)	0.0016 (13)	0.0139 (10)	0.0017 (12)
C12	0.0649 (19)	0.073 (3)	0.0406 (15)	0.0098 (19)	0.0047 (14)	−0.0010 (15)
C13	0.066 (2)	0.058 (2)	0.079 (2)	0.0158 (19)	0.0098 (18)	0.014 (2)

### Geometric parameters (Å, º)

**Table:**

S1—C2	1.740 (3)	C5—H5	0.9300
S1—C4	1.772 (2)	C6—C11	1.401 (4)
N1—C2	1.346 (4)	C6—C7	1.405 (4)
N1—N2	1.358 (4)	C7—C8	1.375 (5)
N1—C3	1.411 (4)	C7—H7	0.9300
N2—C1	1.317 (4)	C8—C9	1.381 (4)
N3—C2	1.291 (4)	C8—H8	0.9300
N3—C1	1.377 (5)	C9—C10	1.387 (4)
O1—C3	1.189 (3)	C9—H9	0.9300
O2—C11	1.373 (3)	C10—C11	1.397 (5)
O2—C12	1.444 (4)	C12—H12A	0.9600
O3—C10	1.350 (4)	C12—H12B	0.9600
O3—C13	1.415 (5)	C12—H12C	0.9600
C1—H1	0.9300	C13—H13A	0.9600
C3—C4	1.489 (4)	C13—H13B	0.9600
C4—C5	1.343 (4)	C13—H13C	0.9600
C5—C6	1.439 (4)		
			
C2—S1—C4	90.02 (15)	C8—C7—H7	119.6
C2—N1—N2	109.7 (2)	C6—C7—H7	119.6
C2—N1—C3	117.8 (3)	C7—C8—C9	120.2 (3)
N2—N1—C3	132.5 (2)	C7—C8—H8	119.9
C1—N2—N1	100.8 (3)	C9—C8—H8	119.9
C2—N3—C1	100.9 (3)	C8—C9—C10	120.8 (3)
C11—O2—C12	116.8 (2)	C8—C9—H9	119.6
C10—O3—C13	118.6 (3)	C10—C9—H9	119.6
N2—C1—N3	116.5 (3)	O3—C10—C9	124.5 (3)
N2—C1—H1	121.8	O3—C10—C11	116.7 (3)
N3—C1—H1	121.8	C9—C10—C11	118.9 (3)
N3—C2—N1	112.1 (3)	O2—C11—C10	121.6 (3)
N3—C2—S1	134.8 (2)	O2—C11—C6	117.2 (3)
N1—C2—S1	113.1 (2)	C10—C11—C6	121.1 (3)
O1—C3—N1	124.1 (3)	O2—C12—H12A	109.5
O1—C3—C4	128.9 (3)	O2—C12—H12B	109.5
N1—C3—C4	107.0 (2)	H12A—C12—H12B	109.5
C5—C4—C3	119.9 (2)	O2—C12—H12C	109.5
C5—C4—S1	128.0 (2)	H12A—C12—H12C	109.5
C3—C4—S1	112.1 (2)	H12B—C12—H12C	109.5
C4—C5—C6	131.1 (2)	O3—C13—H13A	109.5
C4—C5—H5	114.5	O3—C13—H13B	109.5
C6—C5—H5	114.5	H13A—C13—H13B	109.5
C11—C6—C7	118.0 (3)	O3—C13—H13C	109.5
C11—C6—C5	118.2 (2)	H13A—C13—H13C	109.5
C7—C6—C5	123.7 (3)	H13B—C13—H13C	109.5
C8—C7—C6	120.9 (3)		
			
C2—N1—N2—C1	0.1 (3)	C3—C4—C5—C6	−179.9 (3)
C3—N1—N2—C1	−179.0 (3)	S1—C4—C5—C6	−0.1 (5)
N1—N2—C1—N3	0.5 (4)	C4—C5—C6—C11	−179.5 (3)
C2—N3—C1—N2	−0.9 (4)	C4—C5—C6—C7	1.2 (5)
C1—N3—C2—N1	0.9 (4)	C11—C6—C7—C8	0.1 (4)
C1—N3—C2—S1	179.5 (3)	C5—C6—C7—C8	179.4 (3)
N2—N1—C2—N3	−0.7 (4)	C6—C7—C8—C9	−0.9 (4)
C3—N1—C2—N3	178.5 (3)	C7—C8—C9—C10	1.9 (4)
N2—N1—C2—S1	−179.61 (19)	C13—O3—C10—C9	2.4 (5)
C3—N1—C2—S1	−0.4 (3)	C13—O3—C10—C11	−178.2 (3)
C4—S1—C2—N3	−178.2 (4)	C8—C9—C10—O3	177.1 (3)
C4—S1—C2—N1	0.4 (2)	C8—C9—C10—C11	−2.2 (4)
C2—N1—C3—O1	−178.9 (3)	C12—O2—C11—C10	55.9 (4)
N2—N1—C3—O1	0.1 (5)	C12—O2—C11—C6	−128.0 (3)
C2—N1—C3—C4	0.2 (3)	O3—C10—C11—O2	−1.9 (4)
N2—N1—C3—C4	179.1 (3)	C9—C10—C11—O2	177.5 (3)
O1—C3—C4—C5	−1.0 (5)	O3—C10—C11—C6	−177.9 (3)
N1—C3—C4—C5	180.0 (2)	C9—C10—C11—C6	1.5 (4)
O1—C3—C4—S1	179.2 (3)	C7—C6—C11—O2	−176.6 (3)
N1—C3—C4—S1	0.2 (3)	C5—C6—C11—O2	4.1 (4)
C2—S1—C4—C5	179.9 (3)	C7—C6—C11—C10	−0.5 (4)
C2—S1—C4—C3	−0.3 (2)	C5—C6—C11—C10	−179.8 (2)

### Hydrogen-bond geometry (Å, º)

**Table:**

*D*—H···*A*	*D*—H	H···*A*	*D*···*A*	*D*—H···*A*
C9—H9···N2^i^	0.93	2.46	3.375 (4)	167
C8—H8···N3^ii^	0.93	2.60	3.529 (4)	173
C1—H1···N3^iii^	0.93	2.65	3.556 (4)	164

Symmetry codes: (i) *x*+1/2, *y*−3/2, *z*; (ii) −*x*, *y*−1, −*z*; (iii) −*x*−1/2, *y*+1/2, −*z*.

## References

[bb1] Bruker (2002). *SMART*, *SAINT* and *SADABS* Bruker AXS Inc., Madison,Wisconsin, USA.

[bb2] El-Sherif, H. A. H., Mahmoud, A. M., Sarhan, A. A. O., Hozien, Z. A. & Habib, O. M. A. (2006). *J. Sulfur Chem.* **27** ,65–85.

[bb3] Flack, H. D. (1983). *Acta Cryst.* A**39**, 876–881.

[bb4] Gilbertsen, R. B., Chan, K., Schrier, D. J., Guglietta, A., Bornemeier, D. A. & Dyer, R. D. (1999). *J. Med. Chem.* **42**, 1151–1160. 10.1021/jm9805081 10197959

[bb5] Karthikeyan, M. S. (2009). *Eur. J. Med. Chem.* **44**, 827–833. 10.1016/j.ejmech.2008.04.022 18579259

[bb6] Köysal, Y., Işık, Ş., Dog~daş, E., Tozkoparan, B. & Ertan, M. (2004). *Acta Cryst.* C**60**, o356–o357. 10.1107/S010827010400410X 15131391

[bb7] Lesyk, R., Vladzimirska, O., Holota, S., Zaprutko, L. & Gzella, A. (2007). *Eur. J. Med.Chem* **42**, 641–648. 10.1016/j.ejmech.2006.12.006 17303290

[bb8] Martin, L., Rabasseda, X. & Castaner, J. (1999). *Drugs Fut.* **24**, 853–857.

[bb9] Özbey, S., Kendi, E., Tozkoparan, B. & Ertan, M. (1999). *Acta Cryst.* C**55**, 1939–1941.

[bb10] Sheldrick, G. M. (2008). *Acta Cryst.* A**64**, 112–122. 10.1107/S0108767307043930 18156677

[bb11] Tozkoparan, B., Aktayb, G. & Yeşilada, E. (2002). *Il Farmaco*, **57**, 145–152. 10.1016/s0014-827x(01)01195-8 11902657

[bb12] Tozkoparan, B., Gokhan, N., Aktay, G., Yeşilada, E. & Ertan, M. (2000). *Eur. J. Med. Chem* **35**, 743-750. 10.1016/s0223-5234(00)00157-4 10960191

[bb13] Tozkoparan, B., Küpeli, E., Yeşilada, E. & Ertan, M. (2007). *Bioorg. Med. Chem.* **15**, 1808–1814. 10.1016/j.bmc.2006.11.029 17166724

